# Effectiveness of electroacupuncture on anxiety: a systematic review and meta-analysis of randomized controlled trials

**DOI:** 10.3389/fpsyg.2023.1196177

**Published:** 2023-12-19

**Authors:** Wan ki Hong, Yeon Ji Kim, Ye rim Lee, Hye In Jeong, Kyeong Han Kim, Seong-Gyu Ko

**Affiliations:** ^1^College of Korean Medicine, Woosuk University, Jeonju, Republic of Korea; ^2^Department of Preventive Medicine, College of Korean Medicine, Kyung Hee University, Seoul, Republic of Korea; ^3^Department of Preventive Medicine, College of Korean Medicine, Woosuk University, Jeonju, Republic of Korea

**Keywords:** electroacupuncture, anxiety, systematic review, meta-analysis, randomized controlled trials

## Abstract

This systematic review and meta-analysis aimed to comprehensively evaluate the effectiveness of electroacupuncture (EA) for patients with anxiety. Randomized controlled trials (RCTs) on the treatment of anxiety by EA up to November 2022 were searched and collected from nine databases. Hamilton Anxiety Rating Scale (HAMA), self-rating anxiety scale (SAS), and adverse reactions were used as outcome indicators. The quality of relevant articles was evaluated using the Cochrane Collaboration’s risk of bias tool. The quality of evidence for each outcome was classified as “low risk,” “unclear risk,” or “high risk.” RevMan 5.0 was used for data analysis. A total of 633 articles were identified from nine electronic databases; 37 RCTs were included, which measured anxiety changes by using EA alone compared to the control group. For the main outcome, EA significantly reduced the HAMA score [Mean difference (MD):−1.13 (95% CI:−2.55–0.29), I^2^:80%], and the quality of evidence was moderate. EA significantly reduced the SAS score (MD:−3.47 (95% CI,−6.57−−0.36), I^2^:88%), and the quality of evidence was moderate. Our meta-analysis shows that EA reduces HAMA and SAS. This study suggests that EA can relieve anxiety. For various uses, additional research is needed on its effect when combined with other treatments.

**Systematic review registration:**

https://www.crd.york.ac.uk/prospero/display_record.php?RecordID=345658, identifier (CRD42022345658).

## 1 Introduction

Anxiety ranks among the top five conditions individuals treat with medical marijuana in Canada and the United States (Reinarman et al., 2011). Anxiety is a detrimental mental disturbance that includes persistent feelings of apprehension, tension, despair, and distress. These disturbances induce physical symptoms such as tachycardia, nervousness, and inability to relax. It is also frequently associated with complications and disability. Anxiety is different from anxiety disorder. Anxiety disorders are one of the most common mental health conditions (Remes et al., 2016; Amorim et al., 2018). The terminology “Anxiety disorders” comprises several conditions, such as panic disorder, social anxiety disorders, anxiety associated with a medical condition, anxiety induced by substance use, and generalized anxiety disorder. They differ from developmentally normative or stress-induced transient anxiety in persistence and impairment of daily functioning (Craske and Stein, 2016). We targeted anxiety, which is a broader category than anxiety disorders, in order to include more studies.

Anxiety is an emotional response to stimuli that humans perceive as threatening. Anxiety implies responses that affect the psychological and emotional sphere and have physiological and functional consequences (Stamenkovic et al., 2018). Pharmacotherapy and psychotherapy are the conventional treatments for anxiety. Regarding pharmacotherapy, anxiolytics, antidepressants, or monoamine oxidase inhibitors are used, with benzo-diazepines being the most used pharmacological resource as anxiolytics (Remes et al., 2016). However, these pharmacological resources can lead to habituation and cause side effects like chronicity, the need for long-term treatment, and high relapse rates. Due to the seriousness of these effects and the drawbacks of pharmacotherapy, finding an effective treatment with fewer undesirable side effects is a crucial task for modern medicine.

Electroacupuncture (EA), based on traditional Chinese medicine acupuncture, uses an electrical device connected to a needle to send electrical currents to the acupoint, thereby stimulating it (Han et al., 2021). The effect of EA is mediated by beta-endorphin (Ulett et al., 1998). High levels of plasma beta-endorphin may be associated with anxiety (Darko et al., 1992). EA, an improvement of traditional acupuncture, is commonly used to treat chronic pain (Vickers and Linde, 2014; Xiang et al., 2019) because of its safety, efficacy, and fewer side effects. Moreover, EA can also relieve anxiety (Pilkington, 2010), though this evidence is from animal experiments. Numerous studies have shown that EA is well tolerated by patients and is as effective as routine care. EA has displayed anxiety-relief effects in many clinical studies (Han et al., 2021).

The purpose of this study is to describe and critically evaluate the effectiveness of EA in treating anxiety. We provide a review of meta-analyses of research concerning the effectiveness of EA in treating anxiety to complement our study of the randomized clinical trials (RCTs) that have been conducted targeting participants with elevated anxiety levels. Although the previous study (Amorim et al., 2018) provided significant evidence regarding the treatment of anxiety by EA, we searched for RCTs concerning such a treatment to reinforce the research conducted in 2018 (Gao et al., 2020). Additionally, we expanded the scope of the target to anxiety and reinforced the databases or search strategy to collect and analyze more RCTs.

## 2 Materials and methods

### 2.1 Criteria for inclusion and exclusion

#### 2.1.1 Study types

RCTs were included. We excluded crossover studies to reduce the risk of potential bias. There were no limitations regarding the publication language of the study.

#### 2.1.2 Participant types

Participants in all groups included people with disease, menopause, addiction, and the healthy. There were no limitations regarding the sex, race, and nationality of the participants. Additionally, we included anxiety but excluded anxiety disorders.

#### 2.1.3 Intervention types and controls

For treatment interventions, studies using EA therapies were included. However, studies that combined EA with other treatments or did not clearly specify anxiety measures were excluded. Control interventions included no treatment or sham acupuncture or other therapies, such as psychosocial interventions, pharmacological interventions, and other conventional interventions.

#### 2.1.4 Outcomes measures

All studies had to use an established rating scale or other effective measures to access the degree of anxiety. The primary outcomes are SAS (Dunstan and Scott, 2020), HAMA (Thompson, 2015). The SAS is a 20-item self-report assessment device built to measure anxiety levels. The total raw scores range from 20 to 80 (20–44: Normal Range, 45–59: Mild to Moderate Anxiety Levels, 60–74: Marked to Severe Anxiety Levels, 75 and above: Extreme Anxiety Levels). HAMA is a psychological questionnaire used by clinicians to rate the se-verity of a patient’s anxiety. The secondary outcomes are (1) state–trait anxiety inventory (STAI), (2)Beck Anxiety Inventories (BAI), (3) Symptom Checklist-90-Revised (SCL-90), (4) Hospital Anxiety and Depression Scale (HADS), (5) Generalized Anxiety Disorder questionnaire of 7 items (GAD-7), (6) VAS (anxiety scores), (7) HRSD (Hamilton Rating Scale for Depression), (8) CAS, (9) PAC-QOL (Patient assessment of constipation quality of life).

### 2.2 Literature searches

We comprehensively searched the following English, Korean and Chinese electronic databases from their inception date to 11 July 2022: Medline (via PubMed), EMBASE (via Elsevier), Cochrane Central Register of Controlled Trials, Science ON, Korean Studies Information Service System, Research Information Sharing Service, Oriental Medicine Advanced Searching Integrated System, China National Knowledge Infrastructure and American Psychological Association PsycArticles. We additionally reviewed the reference lists of the relevant studies to include any potentially relevant studies. We included both the broad term “anxiety [all fields]” AND “Electroacupuncture [all fields]” AND “Randomized Controlled Trials” [all fields]. The detailed search strategies for each database and search results are presented in Supplementary Table. Results were limited to “human” studies. Searches were performed without restriction by year.

### 2.3 Data selection

After removing duplicates, the titles and abstracts of the articles were reviewed for first inclusion. For the studies included after the initial screening, the full texts were retrieved and reviewed for final inclusion.

### 2.4 Data extraction

For the studies finally included, we extracted the following information using a standardized, pilot-tested Excel form: first author’s name, publication year, sample size, details of participants, intervention (acupoints, frequency, duration), control, outcomes, results.

Study selection and data extraction were independently conducted by three researchers (WK, YJ, and YR), and any disagreement was resolved by discussions with the other researchers (HI). If the data were ambiguous or insufficient, we contacted the authors of the included studies via e-mail if possible.

### 2.5 Quality/risk of bias assessment of included studies

We evaluated the methodological quality of the included studies using the Cochrane Collaboration’s risk of bias tool including items of random sequence generation, allocation concealment, blinding of participants and personnel, blinding of outcome assessors, completeness of outcome data, selective reporting, other potential threats to validity. Three researchers (WK, YJ, and YR) independently conducted the risk of bias assessment, and a consensus was reached through discussions with other researcher (HI) if there were disagreements. We classified each item as “low risk,” “unclear risk,” or “high risk.”

### 2.6 Data analysis

If sufficient studies were selected, a meta-analysis was conducted using the mean difference (MD) for continuous variables as effect estimates. The data was analyzed using the Cochrane Review Manager software (RevMan 5.0). Statistical heterogeneity of the RCTs was evaluated using I^2^ statistics and its 95% CI, where I^2^ > 50% or *p* < 0.05 indicated significant heterogeneity. Subgroup analyses were performed for anxiety symptoms to explore potential factors that contributed to the heterogeneity.

## 3 Results

**Figure 1:**
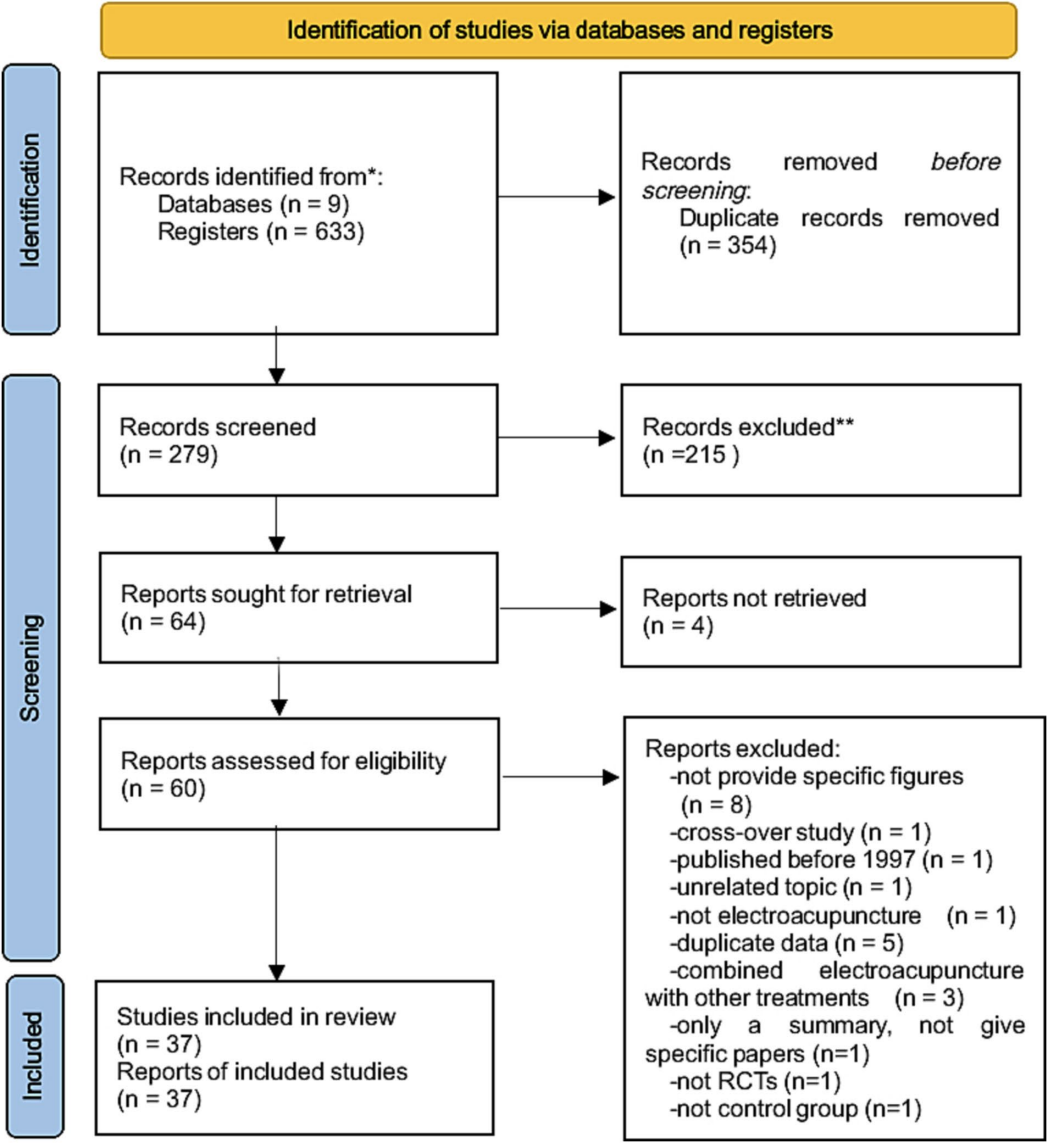
Flow chart showing the number of studies included and excluded from the systematic review.

A total of 633 articles were initially identified from 9 electronic databases. After removing 354 duplicate records, we screened 279 records based on the titles and abstracts, and examined 64 eligible reports further for retrieval. Among these, full-texts were not available for 4 of them. A full-text review was conducted for the remaining 60 articles. After excluding 20 articles for the following reasons: 8 studies did not provide specific figures, 1 study did not use EA, 1 study was a cross-over study, 1 study was not an RCT, 1 study did not include a control group which was inconsistent with our intentions, 1 study only provided a summary but not specific papers, 1 study was unrelated to the topic, 5 studies contained duplicate data, and 4 studies combined EA with other treatments. Finally, a total of 37 studies were included in our analysis (Figure 1).

### 3.1 Study characteristics

The articles included in the review were published in English, Korean, and Chinese between the start date and 11 July 2022, with a variety of patient conditions represented. The most frequently studied conditions were insomnia (4 studies) and depression (3 studies), followed by functional dyspepsia, polycystic ovary syndrome, irritable bowel syndrome, functional constipation, and healthy participants (2 studies each). Other conditions were studied only once. The EA group utilized a total of 68 acupuncture points, with PC6 used most frequently (19 times), followed by GV20 (18 times) and SP6 (15 times). HT7 was used 12 times, while ST36 and LR3 were used 10 and 9 times, respectively. The electricity frequency categories in the EA group were Low (2-5 Hz) in 19 studies, L-H (the range overlapping L and H) and L-M in 6 studies each. The control group was most commonly treated with SA (sham acupuncture) or drugs, with 12 studies each. Psychological interventions were used in 3 studies, while moxibustion and NA were used in 2 studies each. The most commonly used outcome measures were HAMA and SAS (11 studies each), followed by HADS (5 studies), while other measures were used only once or twice (Table 1).

**Table 1:** Summary of the studies included in the review.

Study	Participants	Sample size (EA/CT)	Intervention				Control	Outcomes	Outcome (pre → pro, χ ± s)		Result (O/△)
			Acupoint(s)	Frequency (Hz)	Type	Duration (min, times/week, total)			Intervention	Control	
Tong et al. (2022) ①	Conservative breast surgery	36/34	HT7,PC6, EX-HN3, DU20	2	L	pre-op, 30 min, 3 t/w, 8 weeks	NA	SAS	47.92 ± 4.05 → 41.14 ± 4.77	46.06 ± 5.08 → 49.91 ± 5.27	O
Tong et al. (2022) ②		36/34				pre-op and intra-op, 30 min, 3 t/w, 8 weeks			47.78 ± 4.92 → 41.97 ± 4.10		O
Yin et al. (2022)	Insomnia	83/83	GV20, GV29	2	L	30 min, 3 t/w, 8 weeks	SA	SAS	2.9*	0.14*	O
Xu et al. (2022) ①	Functional dyspepsia	24/19	ST25, BL25	2	L	30 min, 3–5 t/w, 4 weeks	Medication (loperamide hydrochloride capsule 2 mg)	SAS	31.63 → 25.41	32.47 → 32.87	O
Xu et al. (2022) ②		25/19	ST25, BL25	50	M	30 min, 3–5 t/w, 4 weeks			31.85 → 28.9	32.47 → 32.87	O
Bakacak et al. (2020)	HSG	36/37	H7, Du20, Liv-3, P-6, HT7, PC6, LI4, LI10, SP6, LR3, ST36, GB26, CP15, ST28 and Ren-4 points	1–20	L-M	before HSG 20 min	NA	STAI-S	43 → 32	44 → 41	O
Xing et al. (2020)	Insomnia	30/30	DU20, EX-HN1, EX-HN22, SP6, HT7, PC6, BL62, KI6	2, 100	L-H	30 min, 3 t/w, 4 weeks	CBT	HAMA	11.42 ± 4.23 → 9.52 ± 4.02	11.81 ± 5.10 → 9.41 ± 4.73	△
Liu et al. (2020)	PSAD	35/32	GV20, GV16. BL15, HT7	20	M	20 min, e.o.d, 3 t/w, 4 weeks	Medication (Paroxetine, 10–20 mg)	HAMA	22.20 ± 4.50 → 10.77 ± 3.45	21.91 ± 3.83 → 11.94 ± 2.85	O
Wang et al. (2019)	PCOS	23/20	CV3, CV6, ST29, SP6, SP9, LI4, GV20. The second points: ST25, ST29, CV3, CV6, LR3, PC6, GV20	2	L	30 min, 2 t/w, 16 weeks	SA	SAS	38 → 35	42 → 43	O
Yinjie et al. (2018)	Spinal cord injury	25/25	GV points, back-shu points	5	L	20 min, 1 t/day, 2 month	Conventional needling	HAMA	19.16 ± 6.55 → 13.98 ± 5.64	20.52 ± 7.86 → 16.65 ± 8.70	O
Zeng et al. (2018)	MA addiction	31/33	T5, L2, PC6, HT7, ST36, SP6	2	L	3 t/w, 4 weeks	SA	HAMA	23.32 ± 5.06 → 5.77 ± 2.53	24.61 ± 5.17 → 8.79 ± 4.46	O
Teoh et al. (2018)	Diagnostic EUS	64/64	LI4, PC6, ST36	2	L	45 min, before op, during op	SA	VAS	4.4 → 1.7	4.3 → 5.1	O
Hui et al. (2017)	IAD	39/36	GV20 EX-HN1, LI4, PC6, LR3, SP6	10–100	L-H	30 min, e.o.d for 10 turns, 2 course	PI	SCL-90	1.5 ± 0.8 → 1.1 ± 0.9	1.5 ± 0.9 → 1.1 ± 0.8	O
Jin et al. (2016)	PCOS	35/33	BL18, CV17, LR14, CV4, ST25, CV4, EX-CA1, SP6, ST36, LR3	20	M	30 min, 10 weeks, total 30 ~ 40 times	Medication (dyne-35,1 tablet)	SCL-90	1.52 ± 0.44 → 1.62 ± 0.46	1.31 ± 0.29 → 1.56 ± 0.42	△
Zhao et al. (2015)	D-IBS	32/30	ST25, ST37	2	L	30 min, qd, 6 t/w, 4 weeks	Moxibustion	HAMA	-^***^		△
Dalamagka et al. (2015) ①	Inguinal hernia	18/18	SP6, ST36, LI4, PC6, BL60, KI3, auricular points Thalamus 26a, Shen-Men55, Lung101	1–2	L	Pre-op 40 min, during surgery, post-op 60 min	SA	STAI	-^***^		O
Dalamagka et al. (2015) ②						Pre-op 40 min, post-op 60 min			-^**^		O
Xiong et al. (2014) ①	Functional constipation	33/34	LI11, ST37	2	L	30 min, 16 times, 4 weeks	Medication (mosapride citrate, 5 mg)	SAS	39.78 ± 8.10 → 37.90 ± 8.22	39.83 ± 10.35 → 40.17 ± 13.01	O
Xiong et al. (2014)②		37/34	LI11, ST37	50	M				40.02 ± 8.01 → 34.49 ± 7.36		O
Dias et al. (2014)	Medical students	30/34	ST36, PC6, GB14, GV20, EX-HN1	2	L	20 min, 6–8 weeks	NA	BAI	15.6 ± 12.7 → 7.9 ± 6.9	12.3 ± 9.1 → 10.8 ± 9.3	O
Mao et al. (2014)	WBC	19/19	at least 4 local points around the joint, at least 4 distant points	2	L	30 min, 10 times, 8 weeks	SA	HADS	−3.5 → −0.7	−1.0 → 0.5	O
Chen and Fan (2013)	GAD	32/31	GV20,EX-HN1,PC6,HT5,KI6,ST36,SP6,LR3	2–15	L-M	30 min, qd, 5 t/w, 6 weeks	Medication (Celite, 10 ~ 20 mg)	HAMA	21.81 ± 3.93 → 11.28 ± 5.72	21.45 ± 4.06 → 10.58 ± 4.84	△
Wang et al. (2012)	E-PTSD	63/64	Ex-HN1, GV20, GV24, GB20	100	H	30 min, e.o.d, 12 weeks	Medication (paroxetine, 20 mg)	HAMA	11.6 ± 5.11 → 2.95 ± 2.85	11.7 ± 5.85 → 3.86 ± 3.15	O
Dias et al. (2012)	Medical students	12/13	ST36, PC6, GB14, GV20, EX-HN1	2	L	20 min, 1 t/w, 8 weeks	NA	BAI	10.2 ± 8.7 → 5.7 ± 2.9	13.3 ± 11.4 → 14.0 ± 13.6	O
Chung et al. (2012)	postpartum depression	5/9	DU20, EX-HN3, EX-HN1, GB15, EX-HN1, GB15, GB8, EX-HN5, ST8, SP6, LR3, HE7, PC6	2	L	30 min, 2 sessions weekly, 4 weeks	SA	HADS	11.0 ± 2.3 → 8.6 ± 3.7	10.3 ± 2.9 → 8.7 ± 4.2	O
Zhu et al. (2011)	IAD	39/36	GV20, EX-HN1, LI4, PC6, LR3, SP6	10–100	L-H	e.o.d, total 20 times	the cognition and behavior therapy	SAS	54.1 ± 10.93 → 44.18 ± 8.85	55.83 ± 9.02 → 47.31 ± 11.56	O
Ping and Songhai (2008)	PSAN	34/33	GV20, GV24, EX-HN3, GV26, LI4, LR3, HT7, PC6	a frequency of 80-100/min	・	30 min, 15 times, OD, 2 courses	Medication (Alprazolam, 0.4–0.8 mg)	HAMA	22.31 ± 3.14 → 15.38 ± 3.20	22.27 ± 3.22 → 14.15 ± 3.46	△
								SAS	62.42 ± 7.28 → 51.66 ± 6.57	63.75 ± 6.07 → 48.83 ± 7.13	△
Peng et al. (2008)	Functional dyspepsia	20/20	PC6, ST36	40	M	30 min, qd, 2 weeks	Medication (Cisapride, 10 mg)	SAS	-^***^		O
Gejervall et al. (2005)	IVF	78/80	KI11, ST29, LI10, LI4, ST36, GV20	2–80	L-H	From surgery to recovery	Medication (flunitrazepam 0.5 mg, rectal paracetamol 1 g, alfentanil 0.5 mg)	VAS	28.6 ± 19.8 → 6.5 ± 8.7	30.4 ± 21.2 → 7.2 ± 11.1	△
								STAI	34.8 ± 8.4 → 26.8 ± 5.6	34.3 ± 8.5 → 28.6 ± 7.8	△
Luo et al. (1998)	Depressive	133/108	GV20, EX-HN3	2	L	45 min, 6 t/w, 6 weeks	Medication (amitriptyline, 161 mg)	HRSD	1.35 ± 0.05 → 0.17 ± 0.03	1.24 ± 0.06 → 0.32 ± 0.05	O
Rampes et al. (1997)	AD or -abuse	23/16	HT7	100	H	30 min, weekly, 6 weeks	Counseling	CAS	11 → 4.6	9.2 → 12.0	O
Lee et al. (2020)	Insomnia	49/49	HT7, PC6, BL63, KI4	4	M	30 min, 2–3 t/w, for 4 weeks	SA	HADS	4.59 → 2.63	4.85 → 3.28	O
Yin et al. (2020)	Depressive	27/24	GV20, GV24, GV29, bilateral EX-HN22, HT7, SP6, PC6.	30	M	30 min, e.o.d, 3 t/w, 8 weeks	SA	HAMA	22.33 ± 8.76 → 10.53 ± 7.53	24.87 ± 8.42 → 20.90 ± 8.17	O
Zhao et al. (2018)	C-IBS + healthy (7)	30/30	ST25, ST37 bilaterally	2	L	30 min, OD, 6 t/w, 4 weeks	Moxibustion	HAMA	-^***^		O
Xu et al. (2020)	Functional constipation	30/30	LI11, ST37 bilaterally	2/50	L-H	30 min, 3–5 t/w, 4 weeks	Medication (mosapride citrate, 5 mg)	SAS	-^**^		O
Li et al. (2020)	PMI	42/42	GV20, GV24, GV29, CV6, CV4, bilateral EX-HN22, SP6, HT7, GV4, BL23, KI3, KI7	2.5	L	30 min, 1–3 t/w, 8 weeks	SA	SAS	46.43 ± 4.45 → 44.98 ± 3.85	47.43 ± 6.66 → 48.10 ± 6.04	O
Caiyuzhu et al. (2017)	Manopasual	25/25	CV4, EX-CA1, ST25, SP6	10, 50	M-H	30 min, e.o.d, 3 t/w, 8 weeks	SA	SAS	38.72 ± 5.37 → 32.67 ± 4.46	39.50 ± 6.45 → 31.89 ± 5.05	△
Zhang et al. (2018)	Depressive	30/30	GV20, GV24, PC6, HT7, SP6, LR3	10, 50	M-H	QD, 30 min, 5 days/week, 8 weeks	Music	HAMA	18.07 ± 2.74 → 10.60 ± 1.94	18.03 ± 3.20 → 9.67 ± 3.33	△
Ma et al. (2021)	knee osteoarthritis	38/39	LI10,LI11,TE14,LI14,LU5,TE9	2	L	QD post OP, 20 min, 5 days	Medication (fentanyl, 0.25 μg)	HADS	10.23 ± 3.37 → 9.00	10.58 ± 2.98 → 7.00	△
Kim et al. (2021)	MDD	14/16	GV20, EX-HN3	10	M	20 min, 20 sessions, 8 weeks	SA	STAI (state)	−2.58^*^	−1.25^*^	O
								STAI (trait)	−5.42^*^	−3^*^	O
Yeung et al. (2019)	Long-term b users	72/72	EX-HN1, EX-HN22, GB8, ST8, EX-HN5, GB15, PC6, HT7, SP6, LV3, EX-HN3, GV24, GV20	4	L	30 min	SA	HADS	3.5 → 3.9	3.8 → 4.3	O

EA: electro acupuncture; STAI:state–trait anxiety inventory; PI: psychological intervention; SCL-90: Symptom Checklist-90-Revised;SAS: Self-rating anxiety scale; BAI: the Beck Anxiety Inventories; HADS: Hospital Anxiety and Depression Scale; qd: quaque die (daily);SA: Sham acupuncture; LIS, HIS: Low, High intensity stimulation; Tid: tid ter in die (3 times a day); NI: no intervention; qn: quaque nocte; e.o.d: every other day; ESAS: Edmonton Symptom Assessment System; OD: once a day;SAS: self-rating anxiety scale; BAI: Beck Anxiety Inventory; GAD-7: Generalized Anxiety Disorder questionnaire of 7 items; OASIS: Overall Anxiety Severity and Impairment Scale; CBT: cognitive behavioral therapy; SSRIs: selective serotonin reuptake inhibitors; PCA: patient-controlled sedation and analgesia; VAS: visual analogue scale; OPU: ovum pick up; qd: quaque die (daily) L: Low (2-5 Hz); M: Mid (6-49 Hz); H: High (50-100 Hz); L-H: The range overlaps L and H; ^*^change value; ^**^Graph only, no specific figure; ^***^no graph and specific figure; O: The EA group reduced anxiety values more than the control group; △: The Control group reduced anxiety values more than the EA group.

### 3.2 Quality/risk of bias of included studies

**Figure 2:**
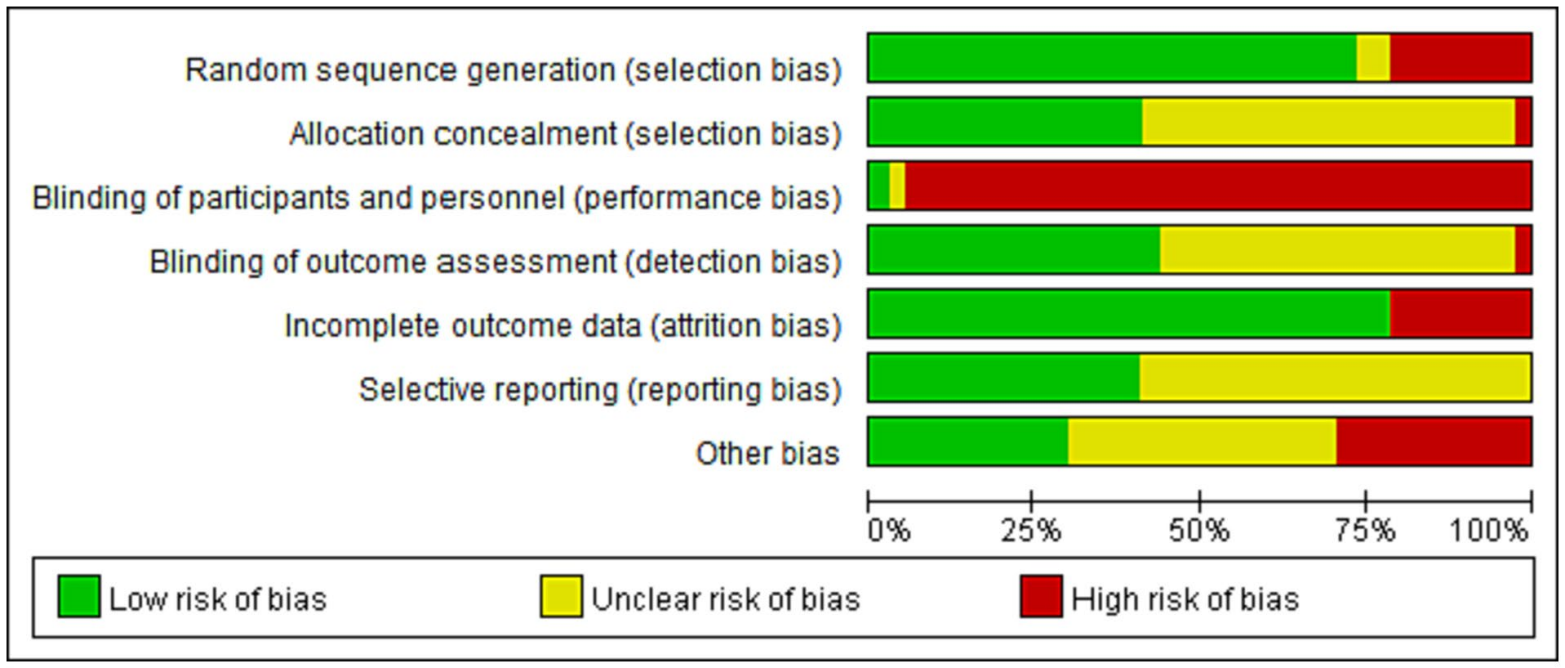
Risk of bias (ROB).

**Figure 3:**
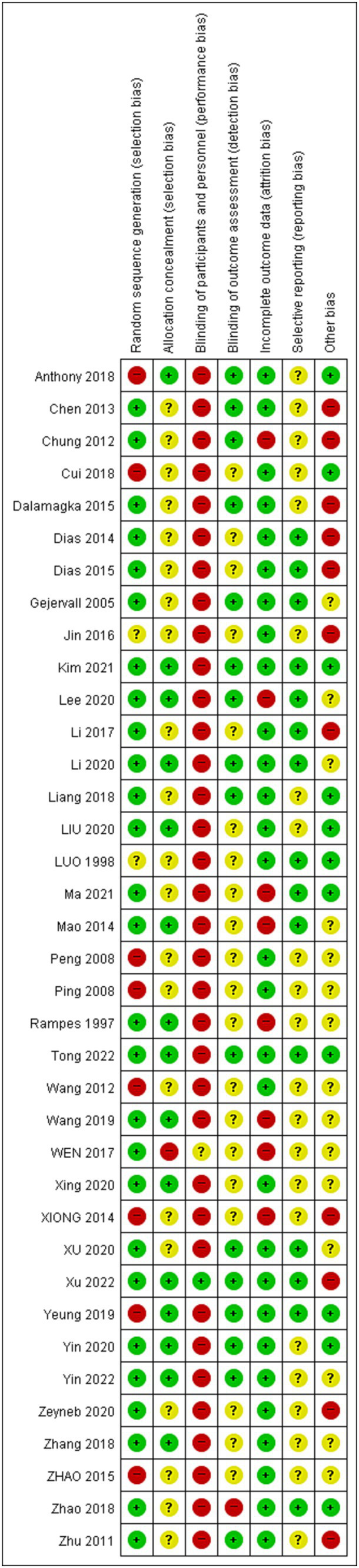
Risk of bias. (graph).

All 27 studies included in the analysis reported their methods for random sequence generation, which included the use of computer programs and random number generators, and were assessed as having a low risk of bias. Among the allocation concealment methods used, 15 studies used sealed envelopes and were assessed as having a low risk of bias, while 21 studies had an uncertain risk due to lack of specific description about blinding of participants and investigators. Thirty-five studies were evaluated as high risk, one was unclear, and only one study was evaluated as having a low risk of blindness. Sixteen RCTs reported detailed outcome assessment of blindness and were rated as having a low risk of bias. Regarding data integrity, all 29 studies provided detailed descriptions, indicating a low risk of bias. All expected outcomes, including adverse events, were reported in the 15 studies, which were assessed as having a low risk of bias. However, the remaining studies either did not document the protocol or did not mention adverse events, leading to high or uncertain risks. There were 11 studies with high-risk sources of bias, 11 with low-risk, and others were unclear. The risk of bias in each trial is presented in Figures 2, 3.

### 3.3 Intervention effects

The studies included in the analysis used EA as the intervention for anxiety in patients with various diseases. The patient populations across the studies were diverse, with three studies focusing on patients with depression and insomnia, and two studies each on patients with constipation, functional indigestion, Internet addiction, polycystic ovary syndrome, and general medical students experiencing anxiety. The remaining studies examined the use of EA for anxiety symptoms independent of any particular disease. Acupuncture points, frequency, and outcome scales varied across the studies. HAMA and SAS were the most frequently used outcome measures, with 11 trials using them. HADS was used in five trials. A meta-analysis was performed on studies using HAMA and SAS as outcome measures, while other studies used different outcome measures.

#### 3.3.1 HAMA

**Figure 4:**
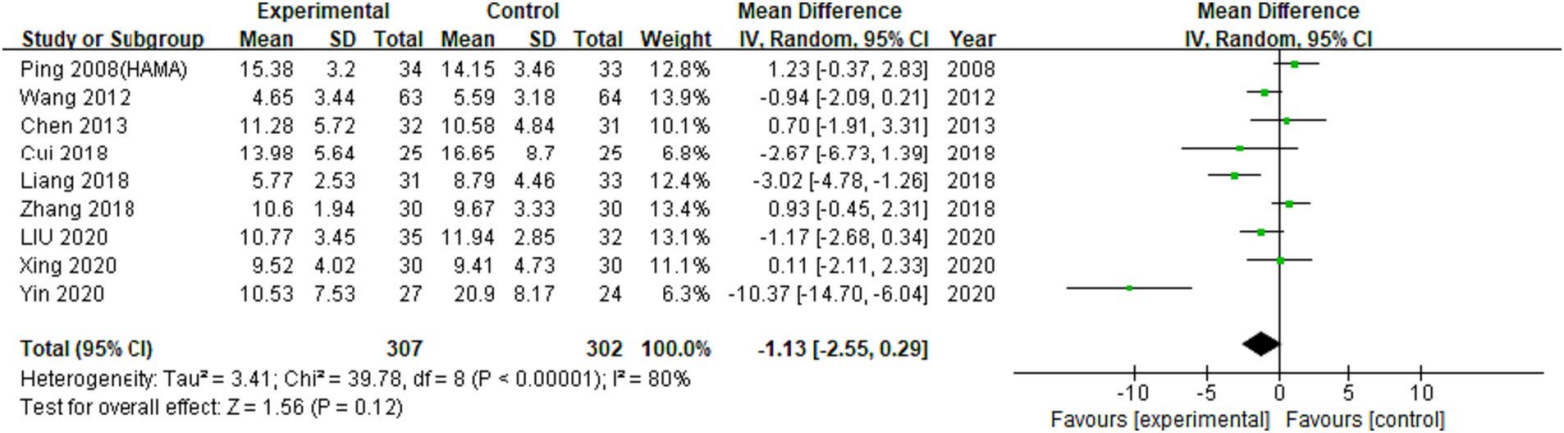
Meta-analysis (HAMA).

A total of 11 RCTs investigated the effect of EA on HAMA scores in our meta-analysis. Meta-analysis of 9 RCTs involving 9 trials with 609 participants were not statistically significant (MD: −1.13 (95% CI −2.55 to 0.29), I^2^:80%, Figure 4).

#### 3.3.2 SAS

**Figure 5:**
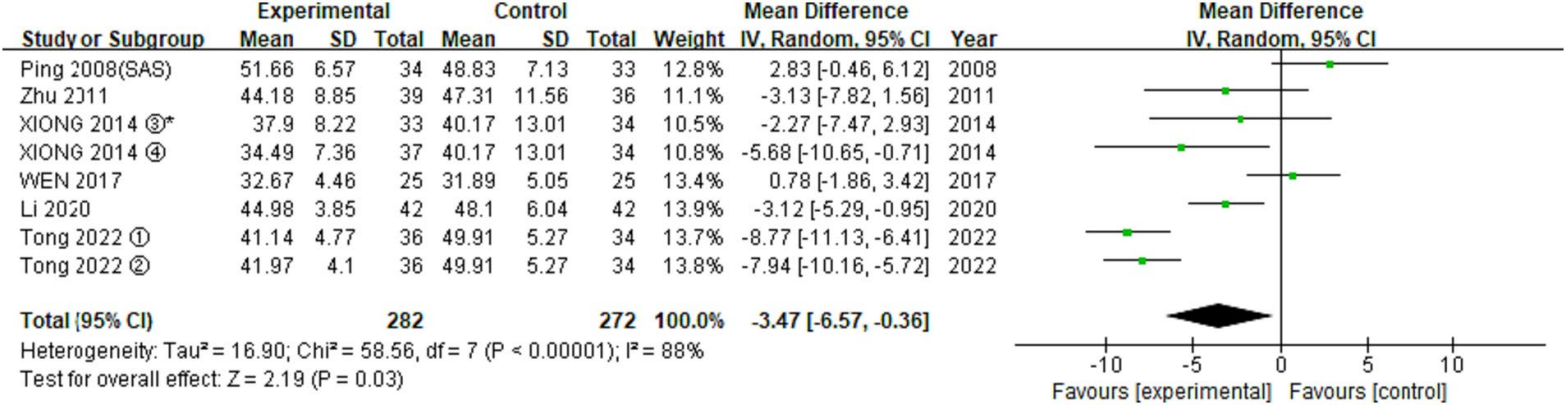
Meta-analysis (SAS).

A total of 11 RCTs investigated the effect of EA on SAS scores in our meta. Meta-analysis of 6 RCTs involving 8 trials with 554 participants revealed significant differences in HAMA score reduction (MD: −3.47 (95% CI −6.57 to −0.36), I^2^:88%, Figure 5) and no significant publication bias.

### 3.4 Subgroup analysis

Since anxiety is a common symptom in many diseases, the primary symptoms of the patients in the studies were not anxiety, but rather other diseases. Therefore, we conducted a meta-analysis using participant status to explore potential differences that may be attributed to variations in patients’ diseases. Among the 37 studies, there were 28 unique participant statuses, indicating little overlap and considerable diversity. The most frequent participant status was insomnia and depression, followed by constipation, diarrhea, Internet addiction, polycystic ovarian syndrome, and two studies involving general patients. We categorized the studies into psychiatric disorders and digestive disorders for the meta-analysis.

#### 3.4.1 Mental symptoms

**Figure 6:**
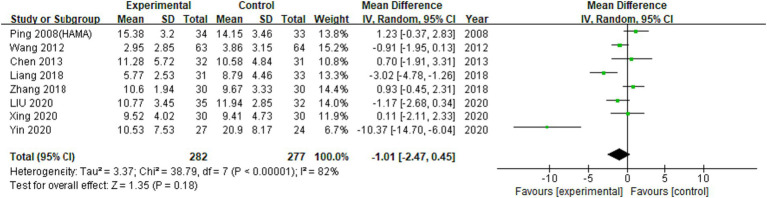
Meta-analysis (Mental symptoms group using HAMA).

A total of 13 RCTs investigated the effects of EA on participants with psychiatric conditions in the meta-analysis. Insomnia, depression, stress disorder, and methamphetamine addiction were included, but Internet addiction was not included (Grant and Chamberlain, 2016). A meta-analysis of 8 RCTs using HAMA outcome measure found results that can infer the tendency that anxiety is effective compared to previous graph results, but were not statistically significant (Figures 4, 5; MD: −1.01 (95% CI −2.47 to 0.45), I^2^:82%, Figure 6).

## 4 Discussion

Anxiety ranks in the top 10 causes of disability worldwide (Remes et al., 2016). Pharmacotherapy and psychotherapy are the conventional treatments for anxiety. However, these pharmacological resources can lead to habituation and cause side effects. Due to its chronicity, high relapse rates, and need for long-term maintenance treatment, there is an urgent need for effective treatment of anxiety with fewer undesirable side effects. Several studies have shown the positive effects of acupuncture on state anxiety (Yang et al., 2021).

This study aimed to update the treatment effect of EA on anxiety by collecting data from RCTs. In this review, we searched several databases to identify comprehensive data sources. The purpose of this review was to provide an overview of previous studies and investigate the effectiveness of EA. Forty-one trials from 37 studies were included in this review, and 20 studies were suitable for meta-analysis. They were mainly conducted in Korea, the United States, and China. The conditions of the patients almost varied without overlapping.

The frequently used acupoint (PC6) was among patients with poststroke onset insomnia and was reported to be effective due to the reduction in sympathetic hyperactivities after acupuncture (Lee et al., 2009). Reduction of sympathetic hypersensitivity was shown to relieve anxiety in this study. The second most used acupoint was GV20. Acupuncture at this point calms the mind and invigorates the brain (Er-Jun et al., 2020). The third and fourth most frequently used acupoints were SP6 and HT7. EA at SP6 and HT7 reduced serum dopamine levels in patients with anxiety, suggesting that EA at SP6 and HT7 could improve the threshold of awakening and regulate abnormal emotions (Li et al., 2022). For the 13 RCTs in the previous paper (Amorim et al., 2018) related to anxiety and acupuncture, PC6 was used seven times, HT7 five times, GV20 three times, and SP6 two times. All four acupuncture points frequently used in our paper were also frequently used in previous papers. Therefore, the four acupoints are often used to relieve anxiety.

For the electric frequency, 19 out of 37 experiments used a low frequency of 2–5 Hz. Two experiments used 100 Hz high frequency alone. In general, in the case of low frequency, beta-endorphin in the brain and enkephalin and dynorphin in the spinal cord are secreted, and it is known that serotonin is involved in high frequency. Anxiety is associated with beta-endorphin, so lower frequencies were used more frequently (Han and Sun, 1990).

The degree of risk of bias (ROB) in other parts is compliant, but the ROB of performance bias is high. This is because the acupuncturist’s blindfold is difficult to implement.

The outcome measures frequently used in this study are divided into two types: using HAMA and SAS to score the acuteness of anxiety level of patients. The results were generally effective, but the heterogeneity was high, so the interpretation should be careful. In previous studies, various outcome indicators were used to measure anxiety. Although a study reported that STAI was the most popular inventory for determining the degree of anxiety during an RCT (Amorim et al., 2018), our analysis confirmed that HAMA and SAS were more frequently used as measures of anxiety to determine the effect of EA.

Previous studies have investigated the relationship between anxiety disorders and acupuncture. We expanded the range of anxiety disorders and checked whether EA was effective on more transient anxiety states. Unlike the existing papers on the relationship be-tween acupuncture and anxiety, we selected studies that only compared the results of the EA and control groups and excluded studies on other treatments. Therefore, the effect of EA on anxiety was observed more clearly.

This review had several limitations. First, the diversity of data could not be secured because it was impossible to research other databases, such as the Japanese database. To overcome this shortcoming, multi-regional studies are required to supply solid clinical outcomes and diverse mechanistic techniques (Guo et al., 2020). Second, the EA parameters, including acupoints and times and frequency of EA, were selected without a consolidated standard in the included studies, which could be a potential source of clinical heterogeneity (Gao et al., 2021). Third, this study regarded EA as the only intervention and did not explore the combined use of other treatment methods for better efficacy. Only the efficacy of EA alone was reviewed; hence, the effect of EA combined with other treatments is unknown. Further research is needed in this area (Zhou et al., 2022). Fourth, studies with a high methodological quality were relatively limited, based on the Cochrane Collaboration’s assessment tool. Critical information related to allocation concealment, blinding methods of participants, and personnel and outcome assessment was missing in most of the included trials (Guo et al., 2020). Due to the active manipulation of EA, most clinical studies could not be completely blinded.

However, unlike previous studies, the scope of the subjects was not limited to anxiety disorders with long-term symptoms; it included many studies by broadening the scope of subject RCTs to include transient anxiety emotions. As EA is effective for anxiety, it is expected to be used as an adjunct therapy in surgery and as a direct anxiety treatment. Since we only targeted RCTs in which electroacupuncture was treated as a single treatment, the relationship between electroacupuncture and anxiety could be identified.

## 5 Conclusion

The information gathered in this systematic review leads to observation and conclusion that is that different methodologies (different acupoints, frequency type, duration) lead to similar results which are decreased levels of anxiety. Our meta-analysis shows that EA reduces HAMA and SAS. The evidences from this study suggest that EA can help in relieving anxiety.

## Data availability statement

The original contributions presented in the study are included in the article/Supplementary material, further inquiries can be directed to the corresponding author.

## Author contributions

WH, YK, and YL: conceptualization, methodology, formal analysis, investigation, data curation, writing—original draft preparation, and visualization. WH: software. HJ: writing—review and editing. HJ, KK, and SK: supervision, project administration. S-G.K: Writing – review & editing, Supervision, Project administration, and Funding acquisition. All authors contributed to the article and approved the submitted version.

## References

[ref1] Amorim D. Amado J. Brito I. Fiuza S. M. Amorim N. Costeira C. . (2018). Acupuncture and electroacupuncture for anxiety disorders: a systematic review of the clinical research. Complement. Ther. Clin. Pract. 31, 31–37. doi: 10.1016/j.ctcp.2018.01.008, PMID: 29705474

[ref2] Bakacak Z. Demirel A. Bakacak M. Urfalıoğlu A. Yaylalı A. Boran Ö. F. . (2020). A randomized pilot study of electro-acupuncture treatment for hysterosalpingography pain relief and related anxiety. J. Turkish Soc. Obst. Gynecol. 17, 253–258. doi: 10.4274/tjod.galenos.2020.66592, PMID: 33343971 PMC7731605

[ref3] Caiyuzhu W. Yafei L. Xiaoli P. Zhen M. Li Z. Hongxing Z. (2017). Manual acupuncture versus electroacupuncture for menopausal syndrome: a randomized controlled trial. Zhongguo Zhen Jiu 37, 491–495. doi: 10.13703/j.0255-2930.2017.05.011, PMID: 29231609

[ref4] Chen Y. Fan Z. (2013). Randomized control study on combination of ear acupoint bloodletting and electroacupuncture for treating mild to moderate generalized anxiety disorder. J. Mod. Med. Health 13, 1928–1930. doi: 10.3969/j.issn.1009-5519.2013.13.004

[ref5] Chung K.-F. Yeung W.-F. Zhang Z.-J. Yung K.-P. Man S.-C. Lee C.-P. . (2012). Randomized non-invasive sham-controlled pilot trial of electroacupuncture for postpartum depression. J. Affect. Disord. 142, 115–121. doi: 10.1016/j.jad.2012.04.008, PMID: 22840621

[ref6] Craske M. G. Stein M. B. (2016). Anxiety. Lancet 388, 3048–3059. doi: 10.1016/S0140-6736(16)30381-6 27349358

[ref7] Dalamagka M. Mavrommatis C. Grosomanidis V. Karakoulas K. Vasilakos D. Samara M. . (2015). Postoperative analgesia after low-frequency electroacupuncture as adjunctive treatment in inguinal hernia surgery with abdominal wall mesh reconstruction. Acupunct. Med. 33, 360–367. doi: 10.1136/acupmed-2014-010689, PMID: 26040491

[ref8] Darko D. F. Risch S. C. Gillin J. C. Golshan S. (1992). Association of beta-endorphin with specific clinical symptoms of depression. Am. J. Psychiatr. 149, 1162–1167. doi: 10.1176/ajp.149.9.1162, PMID: 1503128

[ref9] Dias M. Pagnin D. de Queiroz Pagnin V. Reis R. L. R. Olej B. (2012). Effects of electroacupuncture on stress-related symptoms in medical students: a randomised controlled pilot study. Acupunct. Med. 30, 89–95. doi: 10.1136/acupmed-2011-010082, PMID: 22459648

[ref10] Dias M. Vellarde G. C. Olej B. Teófilo Salgado A. E. de Barros Rezende I. (2014). Effects of electroacupuncture on stress-related symptoms in medical students: a randomised placebo-controlled study. Acupunct. Med. 32, 4–11. doi: 10.1136/acupmed-2013-010408, PMID: 24113152

[ref11] Dunstan D. A. Scott N. (2020). Norms for Zung’s self-rating anxiety scale. BMC Psychiatry 20:90. doi: 10.1186/s12888-019-2427-6, PMID: 32111187 PMC7048044

[ref12] Er-Jun L. Wei-Ling Z. Jian-Bing W. Fu-Gang Z. Ya-Ping B. (2020). Acupuncture combined with cranial electrotherapy stimulation on generalized anxiety disorder: a randomized controlled trial. Zhongguo Zhen Jiu 40, 1187–1190. doi: 10.13703/j.0255-2930.20190917-k0004, PMID: 33788486

[ref13] Gao Y. Wang Y. Zhou J. Hu Z. Shi Y. (2020). Effectiveness of electroacupuncture for simple obesity: a systematic review and Meta-analysis of randomized controlled trials. Evid. Based Complement. Alternat. Med. 2020, 1–14. doi: 10.1155/2020/2367610, PMID: 32714399 PMC7341404

[ref14] Gao X. Zhang Y. Zhang Y. Ku Y. Guo Y. (2021). Electroacupuncture for gastrointestinal function recovery after gynecological surgery: a systematic review and Meta-analysis. Evid. Based Complement. Alternat. Med. 2021, 1–16. doi: 10.1155/2021/8329366, PMID: 34970326 PMC8714373

[ref15] Gejervall A.-L. Stener-Victorin E. Möller A. Janson P. O. Werner C. Bergh C. (2005). Electro-acupuncture versus conventional analgesia: a comparison of pain levels during oocyte aspiration and patients’ experiences of well-being after surgery. Hum. Reprod. 20, 728–735. doi: 10.1093/humrep/deh665, PMID: 15608039

[ref16] Grant J. E. Chamberlain S. R. (2016). Expanding the definition of addiction: DSM-5 vs. ICD-11. CNS Spectr. 21, 300–303. doi: 10.1017/S1092852916000183, PMID: 27151528 PMC5328289

[ref17] Guo Y. Wei W. Chen J. D. (2020). Effects and mechanisms of acupuncture and electroacupuncture for functional dyspepsia: a systematic review. World J. Gastroenterol. 26, 2440–2457. doi: 10.3748/wjg.v26.i19.2440 32476804 PMC7243644

[ref18] Han X. Gao Y. Yin X. Zhang Z. Lao L. Chen Q. . (2021). The mechanism of electroacupuncture for depression on basic research: a systematic review. Chin. Med. 16:10. doi: 10.1186/s13020-020-00421-y, PMID: 33436036 PMC7805231

[ref19] Han J. S. Sun S. L. (1990). Differential release of enkephalin and dynorphin by low and high frequency electroacupuncture in the central nervous system. Acupunct. Sci. Int. J. 1, 19–27.

[ref20] Hui L. Rongjiang J. Kezhu Y. Bo Z. Zhong Z. Ying L. . (2017). Effect of electro-acupuncture combined with psychological intervention on mental symptoms and P50 of auditory evoked potential in patients with internet addiction disorder. J. Tradit. Chin. Med. 37, 43–48. doi: 10.1016/S0254-6272(17)30025-0, PMID: 29956903

[ref21] Jin C. Pang R. Huang J. Jing X. Wu Z. Zhao J. (2016). Impacts on physical and mental health of patients with polycystic ovary syndrome treated with electroacupuncture:a randomized controlled trial. Zhongguo Zhen Jiu 36, 591–595. doi: 10.13703/j.0255-2930.2016.06.009, PMID: 29231453

[ref22] Kim M. Choi E.-J. Kwon O.-J. Park H.-J. Kim A.-R. Seo B.-N. . (2021). Electroacupuncture plus moxibustion for major depressive disorder: a randomized, sham-controlled, pilot clinical trial. Integr. Med. Res. 10:100727. doi: 10.1016/j.imr.2021.100727, PMID: 34307020 PMC8296085

[ref23] Lee S. Y. Baek Y. H. Park S. U. Moon S. K. Park J. M. Kim Y. S. . (2009). Intradermal acupuncture on Shen-Men and Nei-Kuan acupoints improves insomnia in stroke patients by reducing the sympathetic nervous activity: a randomized clinical trial. Am. J. Chin. Med. 37, 1013–1021. doi: 10.1142/S0192415X09007624 19938212

[ref24] Lee B. Kim B.-K. Kim H.-J. Jung I. C. Kim A.-R. Park H.-J. . (2020). Efficacy and safety of electroacupuncture for insomnia disorder: a multicenter, randomized, assessor-blinded, controlled trial. Nat. Sci. Sleep 12, 1145–1159. doi: 10.2147/NSS.S281231, PMID: 33328773 PMC7735782

[ref25] Li S. Wang Z. Wu H. Yue H. Yin P. Zhang W. . (2020). Electroacupuncture versus sham acupuncture for perimenopausal insomnia: a randomized controlled clinical trial. Nat. Sci. Sleep 12, 1201–1213. doi: 10.2147/NSS.S282315, PMID: 33376432 PMC7764880

[ref26] Li D. Xiao-Jun S. Zhong-Wen L. Ling-Xin L. Yi-Hui Z. (2022). Combined use of Shenmen (HT 7) and Sanyinjiao (SP 6) to improve the anxiety and depression in patients with insomnia: a randomized controlled trial. Zhongguo Zhen Jiu 42, 13–17. doi: 10.13703/j.0255-2930.20210113-k0002, PMID: 35025151

[ref27] Liu R. Chen S. Wang J. (2020). electroacupuncture for post-stroke anxiety disorder on the base of the heart-brain theory: a randomized controlled trial从心脑论治电针中风后焦虑障碍:随机对照试验. World J. Acupunc. 30, 23–28. doi: 10.1016/j.wjam.2020.02.008

[ref28] Luo H. Meng F. Jia Y. Zhao X. (1998). Clinical research on the therapeutic effect of the electro-acupuncture treatment in patients with depression. Psychiatry Clin. Neurosci. 52, S338–S340. doi: 10.1111/j.1440-1819.1998.tb03262.x, PMID: 9895187

[ref29] Ma C. Huang H. Lu R. Qin Z. Wang H. Nie Z. . (2021). Effect of crossing acupoints of the same name of hands and feet on pain after total knee arthroplasty. Chin. J. Tissue Eng. Res. 25, 5798–5803. doi: 10.12307/2021.345

[ref30] Mao J. J. Farrar J. T. Bruner D. Zee J. Bowman M. Seluzicki C. . (2014). Electroacupuncture for fatigue, sleep, and psychological distress in breast cancer patients with aromatase inhibitor-related arthralgia: a randomized trial. Cancer 120, 3744–3751. doi: 10.1002/cncr.28917, PMID: 25077452 PMC4239308

[ref31] Peng S.-F. Yang J.-Y. Shi Z.-H. (2008). Electroacupuncture improves gastric motility, autonomic nerve activity and psychological state in patients with functional dyspepsia. World Chin. J. Digestol. 16:4105. doi: 10.11569/wcjd.v16.i36.4105

[ref32] Pilkington K. (2010). Anxiety, depression and acupuncture: a review of the clinical research. Auton. Neurosci. 157, 91–95. doi: 10.1016/j.autneu.2010.04.002 20451469

[ref33] Ping W. Songhai L. (2008). Clinical observation on post-stroke anxiety neurosis treated by acupuncture. J. Tradit. Chin. Med. 28, 186–188. doi: 10.1016/S0254-6272(08)60043-6, PMID: 19004200

[ref34] Rampes H. Pereira S. Mortimer A. Manoharan S. Knowles M. (1997). Does electroacupuncture reduce craving for alcohol? A randomized controlled study. Complement. Ther. Med. 5, 19–26. doi: 10.1016/S0965-2299(97)80085-4

[ref35] Reinarman C. Nunberg H. Lanthier F. Heddleston T. (2011). Who are medical marijuana patients? Population characteristics from nine California assessment clinics. J. Psychoactive Drugs 43, 128–135. doi: 10.1080/02791072.2011.587700 21858958

[ref36] Remes O. Brayne C. van der Linde R. Lafortune L. (2016). A systematic review of reviews on the prevalence of anxiety disorders in adult populations. Brain Behav. 6:e00497. doi: 10.1002/brb3.497, PMID: 27458547 PMC4951626

[ref37] Stamenkovic D. M. Rancic N. K. Latas M. B. Neskovic V. Rondovic G. M. Wu J. D. . (2018). Preoperative anxiety and implications on postoperative recovery: what can we do to change our history. Minerva Anestesiol. 84, 1307–1317. doi: 10.23736/S0375-9393.18.12520-X, PMID: 29624026

[ref38] Teoh A. Y. B. Chong C. C. N. Leung W. W. Chan S. K. C. Tse Y. K. Ng E. K. W. . (2018). Electroacupuncture-reduced sedative and analgesic requirements for diagnostic EUS: a prospective, randomized, double-blinded, sham-controlled study. Gastrointest. Endosc. 87, 476–485. doi: 10.1016/j.gie.2017.07.029, PMID: 28750840

[ref39] Thompson E. (2015). Hamilton rating scale for anxiety (HAM-A). Occup. Med. 65:601. doi: 10.1093/occmed/kqv054 26370845

[ref40] Tong Q. Liu R. Gao Y. Zhang K. Ma W. Shen W. (2022). Effect of electroacupuncture based on ERAS for preoperative anxiety in breast cancer surgery: a single-center, randomized, controlled trial. Clin. Breast Cancer 22, 724–736. doi: 10.1016/j.clbc.2022.04.010, PMID: 35739000

[ref41] Ulett G. A. Han S. Han J. (1998). Electroacupuncture: mechanisms and clinical application. Biol. Psychiatry 44, 129–138. doi: 10.1016/S0006-3223(97)00394-6 9646895

[ref42] Vickers A. J. Linde K. (2014). Acupuncture for chronic pain. JAMA 311, 955–956. doi: 10.1001/jama.2013.285478, PMID: 24595780 PMC4036643

[ref43] Wang Z. Dong H. Wang Q. Zhang L. Wu X. Zhou Z. . (2019). Effects of electroacupuncture on anxiety and depression in unmarried patients with polycystic ovarian syndrome: secondary analysis of a pilot randomised controlled trial. Acupunct. Med. 37, 40–46. doi: 10.1136/acupmed-2017-011615, PMID: 30843421

[ref44] Wang Y. Hu Y. Wang W. Pang R. Zhang A. (2012). Clinical studies on treatment of earthquake-caused posttraumatic stress disorder using electroacupuncture. Evid. Based Complement. Alternat. Med. 2012, 1–7. doi: 10.1155/2012/431279, PMID: 23049609 PMC3462425

[ref45] Xiang X. Wang S. Shao F. Fang J. Xu Y. Wang W. . (2019). Electroacupuncture stimulation alleviates CFA-induced inflammatory pain via suppressing P2X3 expression. Int. J. Mol. Sci. 20:3248. doi: 10.3390/ijms20133248, PMID: 31269659 PMC6651287

[ref46] Xing J. Wu X. Liu H. Wang J. Jiang S. Lozada A. . (2020). Effects of electroacupuncture therapy and cognitive behavioral therapy in chronic insomnia: a randomized controlled study. Evid. Based Complement. Alternat. Med. 2020, 1–12. doi: 10.1155/2020/5630130, PMID: 32256651 PMC7106874

[ref47] Xiong F. Wang Y. Li S. Tian M. Zheng C. Huang G. (2014). Clinical study of electro-acupuncture treatment with different intensities for functional constipation patients. J. Huazhong Univ. Sci. Technolog. Med. Sci. 34, 775–781. doi: 10.1007/s11596-014-1351-8 25318892

[ref48] Xu X. Zhang M. Wu X. Xu S. Wang W. Zheng C. . (2020). Efficacy of electro-acupuncture in treatment of functional constipation: a randomized controlled trial. Curr. Med. Sci. 40, 363–371. doi: 10.1007/s11596-020-2188-y 32337698

[ref49] Xu X. Zhang M. Wu X. Zheng C. Huang G. (2022). The effect of electroacupuncture treatment with different intensities for functional diarrhea: a randomized controlled trial. Evid. Based Complement. Alternat. Med. 2022, 1–8. doi: 10.1155/2022/2564979, PMID: 35027932 PMC8752238

[ref50] Yang X. Yang N. Huang F. Ren S. Li Z. (2021). Effectiveness of acupuncture on anxiety disorder: a systematic review and meta-analysis of randomised controlled trials. Ann. General Psychiatry 20:9. doi: 10.1186/s12991-021-00327-5, PMID: 33516258 PMC7847562

[ref51] Yeung W.-F. Chung K.-F. Zhang Z.-J. Zhang S.-P. Chan W.-C. Ng R. M.-K. . (2019). Electroacupuncture for tapering off long-term benzodiazepine use: a randomized controlled trial. J. Psychiatr. Res. 109, 59–67. doi: 10.1016/j.jpsychires.2018.11.015, PMID: 30504097

[ref52] Yin X. Li W. Liang T. Lu B. Yue H. Li S. . (2022). Effect of electroacupuncture on insomnia in patients with depression. JAMA Netw. Open 5:e2220563. doi: 10.1001/jamanetworkopen.2022.20563 35797047 PMC9264041

[ref53] Yin X. Li W. Wu H. Dong B. Ma J. Li S. . (2020). Efficacy of electroacupuncture on treating depression-related insomnia: a randomized controlled trial. Nat. Sci. Sleep 12, 497–508. doi: 10.2147/NSS.S253320, PMID: 32765146 PMC7382580

[ref54] Yinjie C. Xiaojuan S. Qian W. Wenchun W. Anren Z. (2018). The effect of “paraplegic triple needling” combined with rehabilitation training on psychological and daily living ability of patients with spinal cord injury. Zhongguo Zhen Jiu 38, 4833–4839. doi: 10.13703/j.0255-2930.2018.05.009, PMID: 29797912

[ref55] Zeng L. Tao Y. Hou W. Zong L. Yu L. (2018). Electro-acupuncture improves psychiatric symptoms, anxiety and depression in methamphetamine addicts during abstinence. Medicine 97:e11905. doi: 10.1097/MD.0000000000011905, PMID: 30142795 PMC6112927

[ref56] Zhang H. Xu N. Li Z. Zhang W. Yi W. (2018). Effects on female depression treated with the combined therapy of acupuncture and the five-element music therapy. Zhongguo Zhen Jiu 38, 1293–1297. doi: 10.13703/j.0255-2930.2018.12.011, PMID: 30672218

[ref57] Zhao J. Lu J. Yin X. Chen X. Chen Y. Tang W. . (2015). Comparison of electroacupuncture and moxibustion on brain-gut function in patients with diarrhea-predominant irritable bowel syndrome: a randomized controlled trial. Chin. J. Integr. Med. 21, 855–865. doi: 10.1007/s11655-015-2049-x, PMID: 25847778

[ref58] Zhao J. Lu J. Yin X. Wu L. Bao C. Chen X. . (2018). Comparison of electroacupuncture and mild-warm moxibustion on brain-gut function in patients with constipation-predominant irritable bowel syndrome: a randomized controlled trial. Chin. J. Integr. Med. 24, 328–335. doi: 10.1007/s11655-018-2838-0, PMID: 29752611

[ref59] Zhou Z. Xu G. Huang L. Tian H. Huang F. Liu Y. . (2022). Effectiveness and safety of Electroacupuncture for depression: a systematic review and meta-analysis. Evid. Based Complement. Alternat. Med. 2022, 1–15. doi: 10.1155/2022/4414113, PMID: 36034955 PMC9410808

[ref60] Zhu T. Li H. Du Y. Zheng Z. Jin R. (2011). Intervention on network craving and encephalofluctuogram in patients with internet addiction disorder:a randomized controlled trial. Chin. Acupunc. Moxibustion 31, 395–399. doi: 10.13703/j.0255-2930.2011.05.006 21692281

