# Supplementary material for: Effectiveness of electroacupuncture on anxiety: a systematic review and meta-analysis of randomized controlled trials
Source: Front Psychol. 2023 Dec 19;14:1196177. doi: 10.3389/fpsyg.2023.1196177 (PMC10764156; doi:10.3389/fpsyg.2023.1196177)
Supplement: Supplementary file 1 [file Data_Sheet_1.docx]

Supplementary Material

Effectiveness of electroacupuncture on anxiety: a systematic review and meta-analysis of randomized controlled trials

Wanki Hong1, Yeon Ji Kim1, Yerim Lee, Hye In Jeong, Kyeong Han Kim*

*** Correspondence:** solip922@hanmail.net; Tel.: +82 10-2777-7610

Table S1. Search strategy in pubmed

| NO. | Search strategy | Item |
| --- | --- | --- |
| #1 | Search "electroacupuncture"[Mesh] | 4643 |
| #2 | Search  (electroacupuncture[Title/Abstract]) OR (electric acupuncture[Title/Abstract]) OR (electrical acupuncture[Title/Abstract]) | 6336 |
| #3 | #1OR#2 | 6889 |
| #4 | Search “anxiety”[Mesh] | 104026 |
| #5 | Search anxiety[Title/Abstract] | 238698 |
| #6 | #4OR#5 | 265279 |
| #7 | Search“Randomized Controlled Trial”[PT] OR “Controlled Clinical Trial”[PT] OR randomized[TIAB] OR placebo[TIAB] OR “Clinical Trials as Topic”[Mesh: noexp] OR randomly[TIAB] OR trial[TI] | 1552214 |
| #8 | #3AND#6AND#7 | 135 |

Table S2. Search strategy in cochrane library

| NO. | Search strategy | Item |
| --- | --- | --- |
| #1 | MeSH descriptor: [Electroacupuncture] explode all trees | 886 |
| #2 | ("electroacupuncture"):ti,ab,kw OR ("electric acupuncture"):ti,ab,kw OR (electrical acupuncture):ti,ab,kw (Word variations have been searched) | 3866 |
| #3 | #1 OR #2 | 3866 |
| #4 | MeSH descriptor: [Anxiety] explode all trees | 9165 |
| #5 | (anxiety):ti,ab,kw | 60614 |
| #6 | #4 OR #5 | 60753 |
| #7 | ("Randomized Controlled Trial"):pt | 553974 |
| #8 | (“Controlled Clinical Trial”):pt OR (randomized):ti,ab,kw OR (placebo):ti,ab,kw OR (randomly):ti,ab,kw OR (trial):ti,ab,kw | 1376651 |
| #9 | #7 OR #8 | 1458594 |
| #10 | #4 AND #5 AND #8 | 209 |

Table S3. Search strategy in embase

| NO. | Search strategy | Item |
| --- | --- | --- |
| #1 | 'electroacupuncture'/exp | 8328 |
| #2 | electroacupuncture:ti,ab,kw OR 'electric acupuncture':ti,ab,kw OR 'electrical acupuncture':ti,ab,kw | 8051 |
| #3 | #1 OR #2 | 9784 |
| #4 | 'anxiety'/exp | 263018 |
| #5 | anxiety:ti,ab,kw | 340776 |
| #6 | #4 OR #5 | 410776 |
| #7 | 'randomized controlled trial'/exp | 720718 |
| #8 | 'randomized controlled trial':it OR 'controlled clinical trial':it OR randomized:ti,ab,kw OR placebo:ti,ab,kw OR randomly:ti,ab,kw OR trial:ab,ti OR 'clinical trials':ti,ab,kw | 2259069 |
| #9 | #7 OR #8 | 2367948 |
| #10 | #3 AND #6 AND #9 | 201 |

Table S4. Search strategy in CNKI

| NO . | Search strategy | Item |
| --- | --- | --- |
| #1 | 电针 | 38500 |
| #2 | 电针治疗 OR 电针刺激 OR 电针疗法 OR 电针穴位疗法 OR 电针组 | 16200 |
| #3 | #1 OR #2  SU=(电针) OR SU=(电针治疗) OR SU=(电针刺激) OR SU=(电针疗法) OR SU=(电针穴位疗法) OR SU=(电针组) | 35500 |
| #4 | SU=(electroacupuncture) OR SU=(electro-acupuncture) OR SU=(electric acupuncture) OR SU=(electrical acupuncture) | 55800 |
| #5 | #3 OR #4  (SU=(电针) OR SU=(电针治疗) OR SU=(电针刺激) OR SU=(电针疗法) OR SU=(电针穴位疗法) OR SU=(电针组)) OR (SU=(electroacupuncture) OR SU=(elecetro-acupuncture) OR SU=(electric acupuncture) OR SU=(electrical acupuncture)) | 36800 |
| #6 | SU=(焦虑) OR SU=(焦虑症) OR SU=(焦虑情绪) OR SU=(焦虑抑郁) | 148300 |
| #7 | Anxiety | 187000 |
| #8 | #6 OR #7  (SU=(焦虑) OR SU=(焦虑症) OR SU=(焦虑情绪) OR SU=(焦虑抑郁)) OR (anxiety) | 148300 |
| #9 | SU=(随机对照试验临床) OR SU=(随机对照试验) OR SU=(对照临床试验) OR SU=(安慰剂) OR SU=(临床试验) OR SU=(试验) | 27936만 |
| #10 | SU=(randomized controlled trial) OR SU=(contolled clinical trial) | 17600 |
| #11 | #9 OR #10  (SU=(随机对照试验临床) OR SU=(随机对照试验) OR SU=(对照临床试验) OR SU=(安慰剂) OR SU=(临床试验) OR SU=(试验)) OR (SU=(randomized controlled trial) OR SU=(contolled clinical trial)) | 27962만 |
| #12 | #5 AND #8 AND #11  (SU=(电针) OR SU=(电针治疗) OR SU=(电针刺激) OR SU=(电针疗法) OR SU=(电针穴位疗法) OR SU=(电针组) OR SU=(electroacupuncture) OR SU=(elecetro-acupuncture) OR SU=(electric acupuncture) OR SU=(electrical acupuncture)) AND (SU=(焦虑) OR SU=(焦虑症) OR SU=(焦虑情绪) OR SU=(焦虑抑郁) OR (anxiety)) AND (SU=(随机对照试验临床) OR SU=(随机对照试验) OR SU=(对照临床试验) OR SU=(安慰剂) OR SU=(临床试验) OR SU=(试验) OR SU=(randomized controlled trial) OR SU=(contolled clinical trial)) | 60 |

Table S5. Search strategy in RISS

| NO | Search strategy | Item |
| --- | --- | --- |
| #1 | 전체: 전침\|전기침\|전기침구\|전기침술 | 4771 |
| #2 | 전체: Electroacupuncture\|electro-acupuncture\|electric acupuncture\|electrical acupuncture | 4073 |
| #3 | #1 OR #2  전침\|전기침\|전기침구\|전기침술 OR Electroacupuncture\|electro-acupuncture\|electric acupuncture\|electrical acupuncture | 8305 |
| #4 | 전체: 불안 | 87,908 |
| #5 | 전체: anxiety | 114,820 |
| #6 | #4 OR #5  불안\|anxiety | 173,976 |
| #7 | 전체: Randomized Trials\|무작위 임상\|무작위 시험\|Randomized Clinical Trials | 120`,392 |
| #8 | #3 AND #6 AND #7  (전침\|전기침\|전기침구\|전기침술\|electroacupuncture\|electro-acupuncture\|electric acupuncture\|electrical acupuncture) AND (불안\|anxiety) AND (Randomized Trials\|무작위 임상\|무작위 시험\|Randomized Clinical Trials) | 4 |

Table S6. Search strategy in KISS

| NO. | Search strategy | Item |
| --- | --- | --- |
| #1 | 전체=”전침”\|”전기침”\|”전기침구”\|”전기침술” \|electroacupuncture\|electro-acupuncture\|”electric acupuncture”\|”electrical acupuncture” | 1059 |
| #2 | 전체=불안\|anxiety | 21,517 |
| #3 | 논문명=”Randomized Trials”\|”Controlled trials”\|”Randomized Clinical Trials”\|무작위 임상\|무작위 시험\|placebo\|Randomized | 9,467 |
| #4 | #1 AND #2 AND #3  전체=”전침”\|”전기침”\|”전기침구”\|”전기침술” \|electroacupuncture\|electro-acupuncture\|”electric acupuncture”\|”electrical acupuncture” AND 전체=불안\|anxiety AND 논문명=”Randomized Trials”\|”Controlled trials”\|”Randomized Clinical Trials”\|무작위 임상\|무작위 시험\|placebo\|Randomized | 0 |

Table S7. Search strategy in OASIS

| NO. | Search strategy | Item |
| --- | --- | --- |
| #1 | ”전침”\|”전기침”\|”전기침구”\|”전기침술” \|electroacupuncture\|electro-acupuncture\|”electric acupuncture”\|”electrical acupuncture” | 607 |
| #2 | 불안\|anxiety | 146 |
| #3 | "Randomized Trials"\|"Controlled trials"\|"Randomized Clinical Trials"\|무작위 임상\|무작위 시험\|placebo\|Randomized | 399 |
| #4 | #1 AND #2 AND #3  (”전침”\|”전기침”\|”전기침구”\|”전기침술” \|electroacupuncture\|electro-acupuncture\|”electric acupuncture”\|”electrical acupuncture”) AND (불안\|anxiety) AND ("Randomized Trials"\|"Controlled trials"\|"Randomized Clinical Trials"\|무작위 임상\|무작위 시험\|placebo\|Randomized) | 0 |

Table S8. Search strategy in APA PsycArticles®

| NO. | Search strategy | Item |
| --- | --- | --- |
| S1 | Mesh(electroacupuncture) OR electroacupuncutre OR "electric acupuncture" OR "electrical acupuncture" | 3 |
| S2 | Mesh(Anxiety) OR anxiety | 55250 |
| S3 | Mesh("Randomized Clinical Trials") OR Mesh("Randomized Controlled Trials") OR ti(placebo) OR ab(placebo) OR "Controlled Clinical Trial" | 2021 |
| S4 | S1 AND S2 AND S3 | 0 |

Table S9. Search strategy in Scienceon.

| NO. | Search strategy | Item |
| --- | --- | --- |
| #1 | 전체=”전침”\|”전기침”\|”전기침구”\|”전기침술” \|electroacupuncture\|electro-acupuncture\|”electric acupuncture”\|”electrical acupuncture” | 62371 |
| #2 | 전체=불안\|anxiety | 310328 |
| #3 | 논문명="Randomized Trials"\|"Controlled trials"\|"Randomized Clinical Trials"\|무작위 임상\|무작위 시험\|placebo\|Randomized | 340899 |
| #4 | #1 AND #2 AND #3  전체=”전침”\|”전기침”\|”전기침구”\|”전기침술” \|electroacupuncture\|electro-acupuncture\|”electric acupuncture”\|”electrical acupuncture” AND 전체=불안\|anxiety AND 논문명="Randomized Trials"\|"Controlled trials"\|"Randomized Clinical Trials"\|무작위 임상\|무작위 시험\|placebo\|Randomized | 246 |
